# Tailoring propagation-invariant topology of optical skyrmions with dielectric metasurfaces

**DOI:** 10.1515/nanoph-2024-0736

**Published:** 2025-03-13

**Authors:** Nilo Mata-Cervera, Zhaoyang Xie, Chi Li, Haoyi Yu, Haoran Ren, Yijie Shen, Stefan A. Maier

**Affiliations:** Centre for Disruptive Photonic Technologies, School of Physical and Mathematical Sciences, 54761 Nanyang Technological University, Singapore 637371, Republic of Singapore; School of Physics and Astronomy, Monash University, Melbourne, VIC, 3800, Australia; School of Electrical and Electronic Engineering, 54761 Nanyang Technological University, Singapore 639798, Republic of Singapore; Department of Physics, Imperial College London, London SW7 2AZ, UK

**Keywords:** structured light, topology; optical skyrmions, metasurfaces

## Abstract

Optical Stokes skyrmions represent an emerging class of structured light characterized by intricate topological polarization textures in the beam’s transverse plane. Traditional methods for generating Stokes skyrmions rely on bulky optical setups, driving significant interests in compact, single-device solutions. However, existing approaches fail to ensure propagation-invariant topology, an imperative requirement for advancing applications in this field. In this paper we address this fundamental challenge with a metasurface design based on structural birefringence and geometric phase which manipulates light in dynamic phase iso-curves, achieving arbitrary co-polarization to cross-polarization conversion while maintaining a constant dynamic phase. This design enables propagation-invariant topological features of optical skyrmions produced by a single generation device. Our framework offers a compact platform for shaping topologically stable optical skyrmions, which may stimulate their applications for long-range optical information transfer.

## 1 Introduction

Optical skyrmions have emerged in recent years as an attractive family of topological structured light [1]. Contrary to conventional beam shaping focusing on tailoring spatial modes, the orbital (OAM) and spin angular momenta (SAM), topological structured light consists on combining these fundamental degrees of freedom to embody different topologies in an optical beam [2], [3]. Stokes skyrmions, a class of full Poincaré beams fulfilling a topological map [4], [5], [6], [7], are the most common category of optical skyrmions, in which the polarization state at each transverse plane spans the entire surface of the Poincaré sphere (PS). Significant research efforts have been done to implement topological textures using different optical fields such as SAM [8], [9], [10], [11], electric field in tightly focused beams [12], [13], evanescent fields [14], [15], [16], energy flow [17], to name a few. However, most of them rely on non-propagating light fields in a tight focus or bounded to plasmonic surfaces, making them unsuitable for long-range information transfer. In contrast, Stokes skyrmions are of paraxial nature and can propagate their topology over arbitrary diffraction distances, offering an attractive platform for optical information processing.

Conventional generation of Stokes skyrmions relies on technologies for vector beam generation, such as combination of spatial light modulators (SLMs), digital micromirror devices (DMDs), beam displacers and wave retarders, making the generation stage remarkably bulky [18], [19], [20], [21]. In this regard, metasurfaces (MS) have revolutionized the field of light manipulation, offering a great platform with unprecedented subwavelength spatial resolution and thickness, remarkable lightweight and multi-functionalities in a single device [22], [23], [24], [25], [26], [27]. Dielectric MSs cover a wide range of the electromagnetic spectrum from UV to infrared [28], [29], the applications of which include ultra-thin and achromatic metalenses [30], [31], [32], beam steering [33], [34], holography [35], [36], [37], polarization control and detection [38], [39], [40], [41], [42], [43], [44], [45], vortex generators [39], [46], [47], [48], [49], to name a few. Recently, dielectric MSs have been used as ultra-compact optical skyrmion generators, the design of which includes geometric and dynamic phase-only modulation, in which different topologies were achieved for different circular polarizations [50], or dynamic phase-only modulation in linear polarization with ultra-compact on-fibre fabrication [51]. Other compact generators, so-called skyrmionic wave-plates [52] and GRIN lenses [53], have been designed based on simultaneous control of structural birefringence and geometric phase. In general, these designs yield complementary amplitude profiles shaped in the co-polarized (CoP) and cross-polarized (CrP) waves due to the spatially-varying birefringence, and a spatially varying wavefront in the CrP wave due to the geometric phase. Although all of the previous methods can generate optical skyrmions at a certain propagation plane, none of them has achieved propagation-invariant topology, the crucial property for practical robust information transfer. The reasons arise from the fact that free-space eigenmodes in both polarization components must be generated in order to achieve a propagation-invariant topology. First, phase-only designs [50], [51] cannot deal with this problem due to the lack of amplitude modulation. On the other hand, although the designs based on spatially-varying birefringence and rotation offer amplitude and phase control [52], [54], the dynamic phase induces an additional wavefront in the CoP wave that cannot be compensated by the geometric phase, disrupting the topology upon propagation.

**Figure 1:**
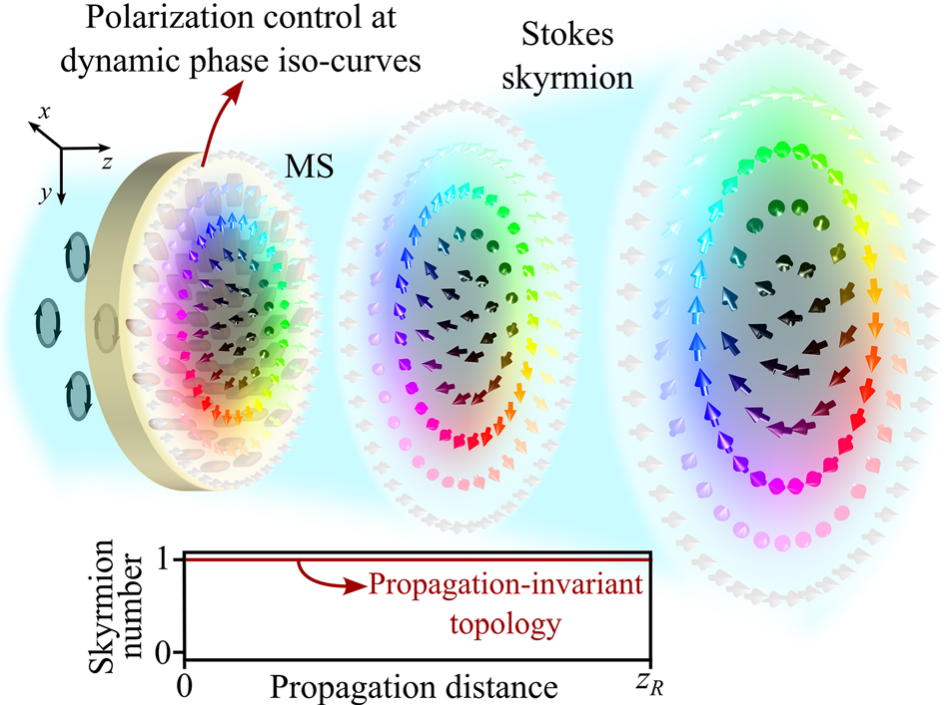
Conceptual figure: Metasurface generating Stokes skyrmions with propagation-invariant topology.

In this paper, we address the challenge of generating Stokes skyrmions with stable topology upon propagation from a single device, which constitutes a step further in realizing compact generators of skyrmionic beams. In the emerging paradigm of topological structured light, distinct optical states exhibit different topologies, making compact generation and topological stability during propagation equally imperative. Here we present a dielectric MS that modulates light at the dynamic phase iso-curves, which allows for spatial control of the structural birefringence and the geometric phase while simultaneously revoking the dynamic phase in both CoP and CrP waves (Figure 1). With this design we have achieved topological invariance of the generated optical skyrmions, featuring a remarkably stable skyrmion number upon diffraction. The lightweight and ultra-thin nature of the designed MSs makes them highly suitable for on-fibre integration, expanding their potential for versatile photonic systems. This compact framework paves the way for possible applications in robust optical communications based on topological structured light.

## 2 Numerical design

Stokes skyrmionic beams can be described as asymmetric counterparts of cylindrical vector beams (CVBs). One can consider a standard spin–orbit coupled CVB in the Laguerre–Gaussian (LG) spatial basis ***E***_CVB_(***r***) = *LG*_*l*,0_(***r***) ***u***_*L*_ + e^i*ζ*^ · *LG*_−*l*,0_(***r***) ***u***_*R*_, and convert it into a skyrmionic beam by introducing spin–orbit symmetry breaking, for instance substituting *LG*_−*l*,0_(***r***) by *LG*_0,0_(***r***):

(1) $$E_{\text{SK}}\left(r\right)=LG_{l,0}\left(r\right)u_{L}+{\text{e}}^{\text{i}\zeta }\cdot LG_{0,0}\left(r\right)u_{R},$$

with *ζ* an arbitrary intermodal phase, $u_{L}=\left(u_{x}+iu_{y}\right)/\sqrt{2}$ and $u_{R}=\left(u_{x}-iu_{y}\right)/\sqrt{2}$ the left- (LCP) and right-handed (RCP) circular polarization basis. Contrary to CVBs which feature all the polarization states contained at the equator of the PS, the selection of *LG*_0,0_(***r***) guarantees that the polarization texture at each transverse plane spans all the surface of the PS from the north (***u***_*L*_) to the south pole (***u***_*L*_). Skyrmionic beams carry nontrivial topologies in their Stokes vector field, whose associated topological number, the skyrmion number

(2) $$N_{\text{SK}}=\frac{1}{4\pi }\underset{\Omega }{\iint }s\cdot \left(\frac{\partial s}{\partial x}\times \frac{\partial s}{\partial y}\right)dxdy,$$

can be stable over arbitrarily long propagation distances. In (2) the spatially varying vector ***s*** = ***s*** (***r***) denotes the inhomogeneous normalized Stokes vector, representing the polarization state at each point of the transverse plane. The Stokes vector is defined as $S=\left(S_{1},S_{2},S_{3}\right)=\left(2Re\left\{\psi_{L}^{⋆}\psi_{R}\right\},-2Im\left\{\psi_{L}^{⋆}\psi_{R}\right\},|\psi_{R}{|}^{2}-|\psi_{L}{|}^{2}\right)$ , and it is normalized with respect to the total intensity $S_{0}=\sqrt{S_{1}^{2}+S_{2}^{2}+S_{3}^{2}}$ , yielding ***s*** = ***S***/*S*_0_. The topological stability of the skyrmion number during propagation requires the spatial profiles of the LCP and RCP components to match with the free-space LG eigenmodes. This ensures that their shapes are preserved, up to a scaling factor, thus maintaining the polarization texture. The challenge arises when trying to generate such eigenmodes from a single optical element, which must control both their intensities and wavefronts. In this paper we construct such profiles using a unitary spin–orbit MS, which will modulate a Gaussian beam *LG*_0,0_(***r***) in one polarization, and a vortex beam *LG*_0,*l*_(***r***) in the orthogonal polarization, with special emphasis in the shape of the wavefronts.

We proceed to describe the response of our MS through its Jones matrix elements, see Supplementary information (SI) S1. For an arbitrary meta-atom shape and orientation, and for left-handed circularly polarized input light ***E***_in_ = ***u***_*L*_, the output polarization state can be expressed as

$$E_{\text{out}}=\frac{1}{2}\left(t_{xx}+t_{yy}\right)u_{L}+\frac{1}{2}\left(t_{xx}-t_{yy}\right)e^{i2\theta }u_{R},$$

where the complex field has been decomposed in its co-polarized (CoP) and cross-polarized (CrP) components, in this case LCP and RCP, respectively. In a simplified description of the transmission we can consider that the Jones matrix is unitary, i.e. $t_{xx}={\text{e}}^{\text{i}\phi_{x}}$ , $t_{yy}={\text{e}}^{\text{i}\phi_{y}}$ . We define the degree of birefringence (DOB) of the MS through the phase difference between the principal axes transmittances:

$$DOB\left(\Delta \phi \right)=\sin\frac{\Delta \phi }{2}=\sin\frac{\phi_{y}-\phi_{x}}{2},$$

which ranges from 0, isotropic plate, to 1, half-wave plate (HWP), for increasing phase difference between 0 and *π*. The transmission through the MS in this simplified unitary approach reads

(3) $$\begin{array}{cc} E_{\text{out}}\left(r\right) & ={\text{e}}^{\text{i}\xi \left(r\right)}\left(\cos\left(\frac{\Delta \phi \left(r\right)}{2}\right)u_{L}\right.\\ & \;+\left.i\sin\left(\frac{\Delta \phi \left(r\right)}{2}\right)e^{i2\theta \left(r\right)}u_{R}\right), \end{array}$$

where we have made explicit that all the magnitudes (*ξ*, Δ*ϕ*, *θ*) are position-dependent. The DOB determines the relative amplitudes between the CoP and CrP waves, while the rotation *θ* defines their relative phases.

A global phase factor $\xi =\left(\phi_{y}+\phi_{x}\right)/2$ , the dynamic phase, appears in both polarization components due to the propagation through the MS. Spatially tailoring the DOB and the rotation allows the generation of arbitrary scalar fields shaped in the CrP component, by simply filtering (absorbing) the CoP light in transmission [55], [56]. As shown in the SI Section S1, full control of the Stokes parameters (*S*_1_, *S*_2_, *S*_3_) can be achieved through the DOB and rotation angle, and the latitude $L=S_{3}$ and azimuth $Z=\arctan\left(S_{2}/S_{1}\right)$ in the PS can be simply expressed as

(4) $$L=\cos\left(\Delta \phi \right),\;Z=\frac{\pi }{2}-2\theta .$$

As seen above, the Stokes parameters do not depend on the dynamic phase $exp\left(i\xi \left(r\right)\right)$ appearing in (3), since it is present simultaneously at both polarization components. However, we note that $exp\left(i\xi \left(r\right)\right)$ is position-dependent, and thus it will induce a spatially-varying wavefront both in the CoP and CrP waves. Of course, the CrP component benefits from an additional degree of freedom provided by the geometric phase, which can counteract the effect of the dynamic phase. However, the CoP component lacks the geometric phase, and thus it will present such a dynamic phase. In general, the shape of $\xi \left(r\right)=\left(\phi_{x}\left(r\right)+\phi_{y}\left(r\right)\right)/2$ will not correspond to a parabolic converging or diverging wavefront, and it will be determined by the spatial distribution of the average refractive indices along *X* and *Y* (SI Section S2). Consequently, even if the CoP amplitude matches that of a Gaussian beam, its wavefront will not, thus its propagation will not correspond to a simple scaling factor.

For our specific goal, the dynamic phase causes a given skyrmion texture at the output plane *z* = *z*_0_ to collapse after propagating a certain distance to *z* = *z*_*f*_, disrupting its topological features. Since no geometric phase approach can be used to overcome this challenge, in this paper we design an MS that compensates the dynamic phase *ξ* using the shape of the meta-atoms instead of the rotation, so that *ξ* remains constant for any value of the DOB for both CoP and CrP waves. In short, our MS performs full CrP/CoP amplitude modulation by the tuning the DOB, and at the same time the amplitude modulation has no effect on the shape of the wavefront of neither the CoP nor the CrP waves.

**Figure 2:**
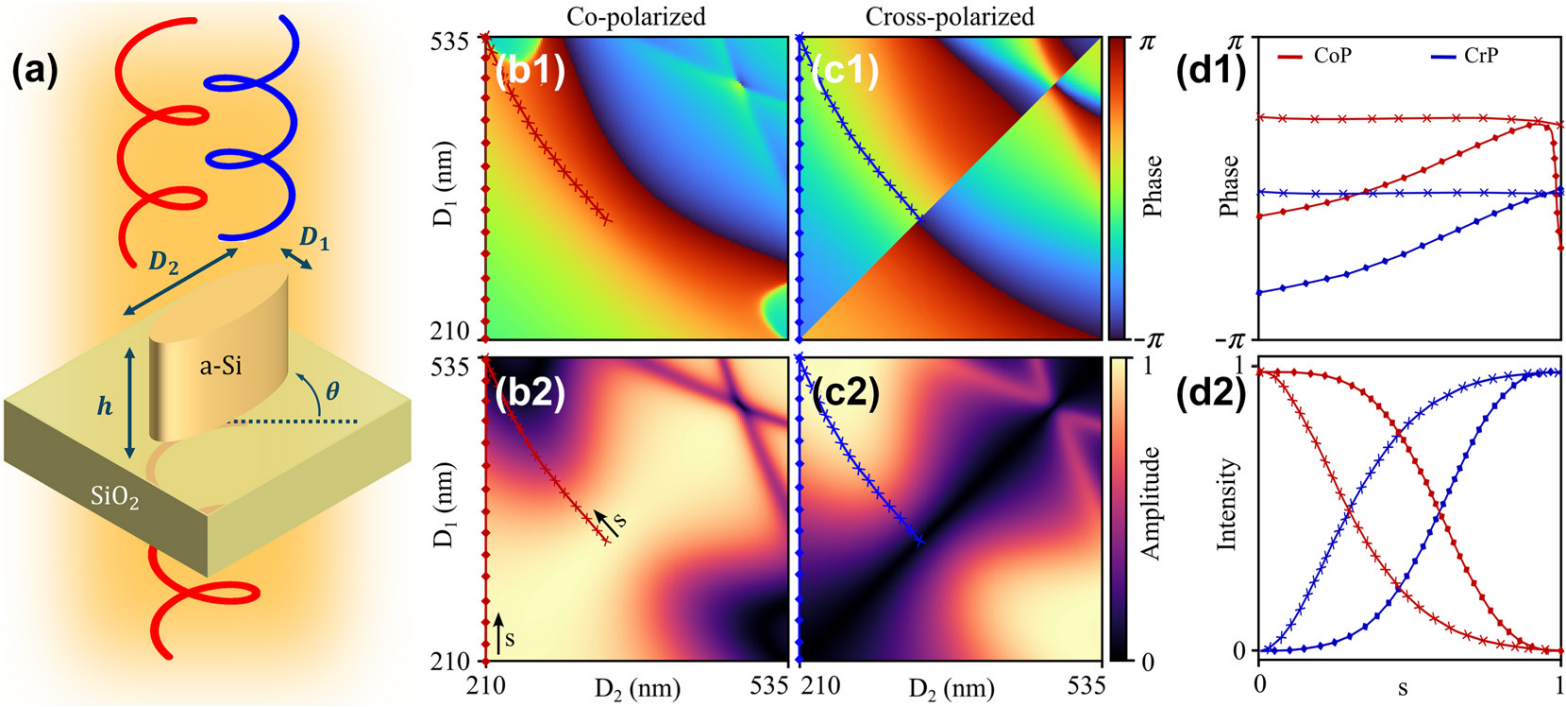
Meta-atom design. (a) Unit cell of the metasurface: *λ* = 1.55 μm, substrate SiO_2_ (*n* = 1.444), elliptical a-Si meta-atoms (*n*_Si_ = 3.505), height *h* = 800 nm, array pitch *P* = 650 nm. Bidimensional library of elliptical meta-atoms: CoP phase (b1) and amplitude (b2), CrP phase (c1) and amplitude (c2). The cross curve depicts the phase iso-curves of *ξ* ≈ − 1.65 rad, and the diamond line represents a slice with fixed *D*_2_ = 210 nm and free *D*_1_. The corresponding phases and intensities for different trajectories in the parametric space are plotted in (d1) and (d2) respectively as a function of an arbitrary parametrization variable *s* (*D*_1_ = *D*_1_(*s*), *D*_2_ = *D*_2_(*s*)), where the blue colour represents CrP waves and the red colour represents CoP components.

A preliminary simulation is carried out using gRCWA [57] at the telecommunications wavelength *λ* = 1.55 μm, the results are shown in Figure 2. As depicted in 2(a) we have chosen a-Si meta-atoms with elliptical cross-section and constant height *h* = 800 nm laying on top of a quartz (SiO_2_) substrate. The unit cell is square with array pitch *P* = 650 nm. At first, we simply sweep the two principal diameters of the ellipses (*D*_1_, *D*_2_) in a range where we can achieve maximal polarization conversion efficiency, from 210 nm to 535 nm. The input light is circularly polarized (LCP) and the output light is decomposed into the CoP (LCP) and CrP (RCP) components. The phase (a1) and amplitude (a2) of the CoP component, and the same (b1) and (b2) for the CrP component are shown. The line *D*_1_ = *D*_2_ delimits the isotropic meta-atoms yielding zero CrP amplitude, while when we move away from it the CrP amplitude increases until it reaches maximum conversion efficiency. As noted in (a2) and (b2), different trajectories in the parametric space (*D*_1_, *D*_2_) can be chosen in order to achieve a full CoP to CrP amplitude modulation, but each trajectory acquires a different dynamic phase, we have highlighted two for comparison. As seen in (d1)-(d2), fixing one diameter (*D*_2_) and tuning the other (*D*_1_) allows for full modulation of CoP–CrP amplitudes (diamond curves), but the CrP and CoP phases will change around *π*/2 over this modulation. For our purpose, we find another trajectory that yields constant phase in the full amplitude modulation, which we denote as phase iso-curves. The phase iso-curves *D*_1_ = *f*(*D*_2_) are fitted using a rational law $D_{1}\left(\mathrm{\mu m}\right)=\alpha /D_{2}^{\beta }+\gamma$ , yielding the optimized set of parameters (*α*, *β*, *γ*) = (0.069683, 1.185457, 0.078194). As shown in (d1)-(d2) the phase iso-curves enable complete CoP–CrP amplitude modulation while keeping a constant phase. This fitting reduces the total number of degrees of freedom in our MS design to two: (*D*_2_, *θ*) and *D*_1_ given by the phase iso-curve *D*_1_ = *f*(*D*_2_).

**Figure 3:**
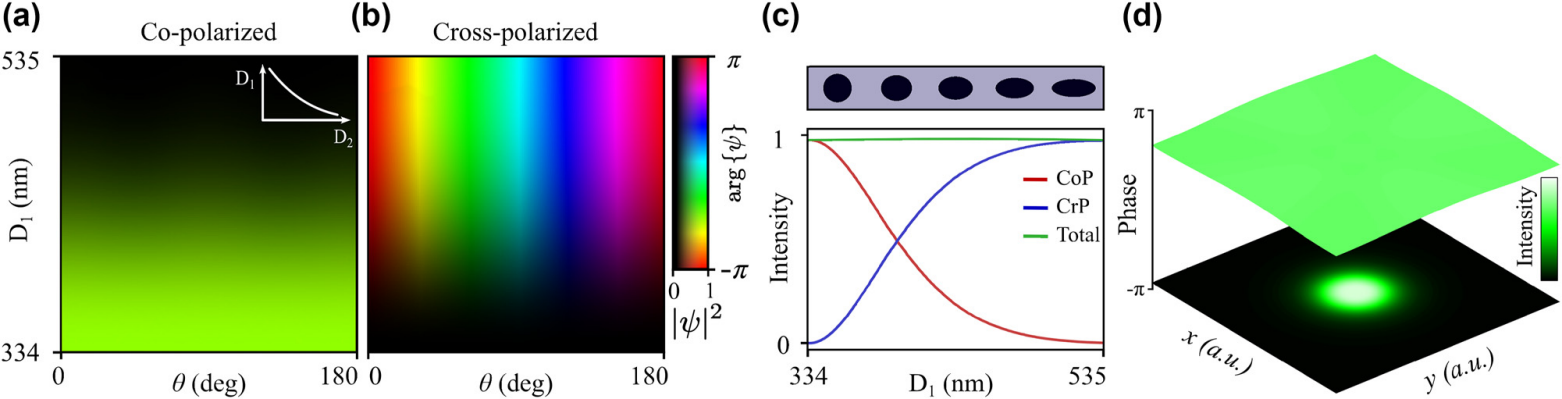
Polarization manipulation at the phase iso-curves: complex amplitudes of the co-polarized (a) and cross-polarized (b) waves represented with a hue-lightness colormap as a function of the first diameter *D*_1_ and the rotation angle *θ*, the second diameter is given by the phase iso-curve equation $D_{1}=\alpha /D_{2}^{\beta }+\gamma$ (inset). The lightness shows the intensity (from 0 to 1) and the hue colour shows the phase (from −*π* to *π*), as depicted in the colorbar. The intensity modulation for *θ* = 0 as a function of *D*_1_ is showed in (c) for the co-polarized (red) and cross-polarized (blue) waves, also plotting the total transmission (green). The inset shows the corresponding transverse shape of the meta-atoms. (d) shows the nearly flat wavefront (surface plot) of a Gaussian beam shaped in the CoP component with data in (a) and its corresponding intensity profile (contour plot). Data: *λ* = 1.55 μm, substrate SiO_2_ (*n* = 1.444), elliptical a-Si meta-atoms (*n*_Si_ = 3.505), height *h* = 800 nm, array pitch *P* = 650 nm.

Following this initial simulation, now we proceed to obtain a definitive meta-atom library for polarization manipulation at the phase iso-curves. The sweep of all the possible diameters and rotation angles (measured counter-clockwise) is shown in Figure 3: the complex amplitudes of the CoP and CrP waves in the parameter space are plotted in (a) and (b) respectively. As expected, the diameter variation yields continuous amplitude modulation with constant phase in both CoP and CrP waves, and the rotation angle provides full phase modulation exclusively in the CrP wave. A slice plot 3(c) for *θ* = 0 shows the CoP/CrP intensity modulation as a function of *D*_1_, keeping the total transmittance around 97 % all over the process. The inset shows the meta-atoms transverse shape within this modulation: higher aspect ratio *D*_2_/*D*_1_ increases the CrP amplitude and carries an additional dynamic phase, which is compensated by a simultaneous reduction of the meta-atoms size along the two principal axes (SI Section S2). In 3(d) we depict an example of a Gaussian intensity profile in the CoP component, featuring a flat wavefront due to the meta-atoms’ geometry.

**Figure 4:**
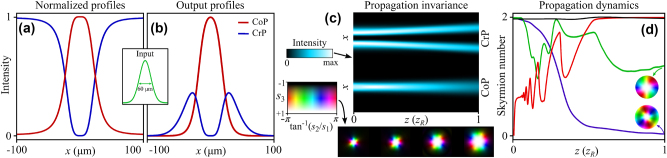
Target and output profiles at the MS: (a) target (normalized) intensity profiles for the CoP and CrP components, the inset shows the input Gaussian illumination over the MS, with $FWHM=w_{0}\sqrt{2\ln2}\approx 60\;\mathrm{\mu m}$ . (b) shows the output intensity profiles after multiplying the target by the input Gaussian beam. (c) shows the evolution of the intensity profiles of the CoP and CrP at *y* = 0 as a function of the propagation distance *z*, the inset shows the Stokes textures for different *z*, yielding an average skyrmion number of ${\bar{N}}_{\text{SK}}\approx 1.97$ . Data: *λ* = 1.55 μm, *D*_MS_ = 200 μm, *w*_0_ = *D*_MS_/4, *w*_1_ = 0.8 ⋅ *w*_0_, *w*_2_ = 0.76 ⋅ *w*_0_, *l* = 2. (d) Shows the evolution of the skyrmion number as a function of the propagation distance for different MS designs: complex amplitude modulation and phase compensation in both CoP and CrP waves (black curve), the same amplitude profiles without any phase compensation (green curve), phase compensation only in the CrP wave with geometric phase (blue curve), and skyrmion Q-plate with no amplitude modulation Δ*ϕ* = *π*/2 (red curve). The inset shows the respective Stokes profile at the final propagation distance. All propagation distances are normalized to the Rayleigh range of the input field $z_{R}=kw_{0}^{2}/2$ .

One more challenge appears when trying to shape propagation-invariant skyrmions with this MS design. We can see from Figure 3(c) that the total transmittance of the MS is constant over all the CrP intensity modulation. Thus, whatever is the polarization texture that we generate, the total intensity will be approximately unitary $\left(|\psi_{L}{|}^{2}+|\psi_{R}{|}^{2}\approx 1\right)$ . However, we would like the output fields to be as much similar to (1) as possible, which does not have unitary total intensity. In our design we start by choosing the spatial modes *ψ*_1_(***r***) = *LG*_0,0_(***r***, *w*_1_) and *ψ*_2_(***r***) = *LG*_*l*,0_(***r***, *w*_2_) (*w*_1_, *w*_2_ denoting the beam waist), and we express the target normalized profiles at the MS as

(5) $$E_{\text{target}}\left(r\right)=\frac{\psi_{1}\left(r\right)u_{L}+\psi_{2}\left(r\right)u_{R}}{\sqrt{|\psi_{1}\left(r\right){|}^{2}+|\psi_{2}\left(r\right){|}^{2}}},$$

satisfying the unitarity condition. The intensity profiles at *y* = 0 for *ψ*_1_(***r***) = *LG*_2,0_ and *ψ*_1_(***r***) = *LG*_0,0_ is depicted in Figure 4(a), showing the profiles that the MS would generate when illuminated by a flat-top. The conversion efficiency (from CoP to CrP) spatially varies from 0 % at the MS center to 100 % at the MS edge. Any experimental deviations of these theoretical values will have a detrimental effect on the measured skyrmion number. Of course, the profiles in Figure 4(a) are not eigenmodes of free-space, but they can be approximately converted into them by simply illuminating the MS with a Gaussian beam *ψ*_0_(***r***) = *LG*_0,0_(***r***, *w*_0_) of appropriate size *w*_0_ [58]. In order to avoid undesired scattering, the intensity of the input beam must decay well-before the edges of the MS, for which we choose the input beam size at around *w* ∼ *D*_MS_/4, with *D*_MS_ the diameter of the MS. The resulting vector beam follows the expression

(6) $$E_{\text{out}}\left(r\right)=E_{\text{target}}\left(r\right)\cdot \psi_{0}\left(r\right),$$

whose intensity profile is plotted in 4(b) again for *y* = 0, showing great resemblance to the free-space LG eigenmodes. To quantitatively confirm the validity of this approach and considering a collimated incident profile *ψ*_0_(***r***), we must find what is the mode decomposition of the resulting beam. The second order moment of the intensity ${\bar{r}}^{2}=\sqrt{2\iint r^{2}|\psi {|}^{2}dxdy/\iint |\psi {|}^{2}dxdy}$ allows us to estimate the “beam waist” $w={\bar{r}}^{2}/\sqrt{1+|l|}$ of an equivalent LG beam, with which we can calculate the overlap integrals. Using the fields in (5) and (6) for *l* = 2, we find that 99.6 % of the power of the CoP wave is in a single mode $LG_{0,0}\left(w\left({\bar{r}}_{1}^{2}\right)\right)$ , while for the CrP wave 97.6 % of the power is in the mode $LG_{l,0}\left(w\left({\bar{r}}_{2}^{2}\right)\right)$ , indicating a very high mode purity for both components. Accordingly, as there is negligible content of modes with radial index, we can expect a propagation invariant evolution of both components as free-space eigenmodes, which is in agreement with the simulation results in Figure 4(c). This evolution guarantees that the Stokes texture (and thus the topology) will not change upon propagation, just expanding as it diffracts (see 4(c) bottom).

To prove the propagation-invariant topology of the output fields, we compute the skyrmion number (2) in propagation for different MS design strategies, the results of which are shown in Figure 4(d). When both CoP and CrP waves are modulated at dynamic phase iso-curves the generated skyrmions feature nearly constant *N*_SK_ (black curve). In contrast, we can compare it with the case where the dynamic phase has not been compensated in the design, as happens for the diamond curves in Figure 2. For instance, if *ξ*(***r***) appears in the CoP wave but it is compensated in the CrP wave through the geometric phase, *N*_SK_ is high at the near field, but it decays with distance due to the non-parabolic converging wavefront in the CoP wave (blue curve). With the same meta-atom design, one could also choose not to compensate the dynamic phase in either CoP or CrP waves, but *N*_SK_ will also not be maintained in propagation (green curve). Finally, if we keep CoP and CrP amplitudes constant, only introducing a vortex phase in the CrP wave (like a Q-plate with Δ*ϕ* = *π*/2), the Skyrmion texture will only appear in the far-field (red curve) [51] once the components of high radial index have diffracted, but will not present skyrmion topology in the near-field. Although our results have been obtained for a topological charge *l* = 2, the same protocol can be applied to achieve higher-order Stokes skyrmions with propagation-invariant topology. In addition, as the generated fields have very high free-space modal purity, they will also preserve their topology at any propagation plane after passing through paraxial optical elements such as lenses.

## 3 Sample fabrication and experimental characterization

**Figure 5:**
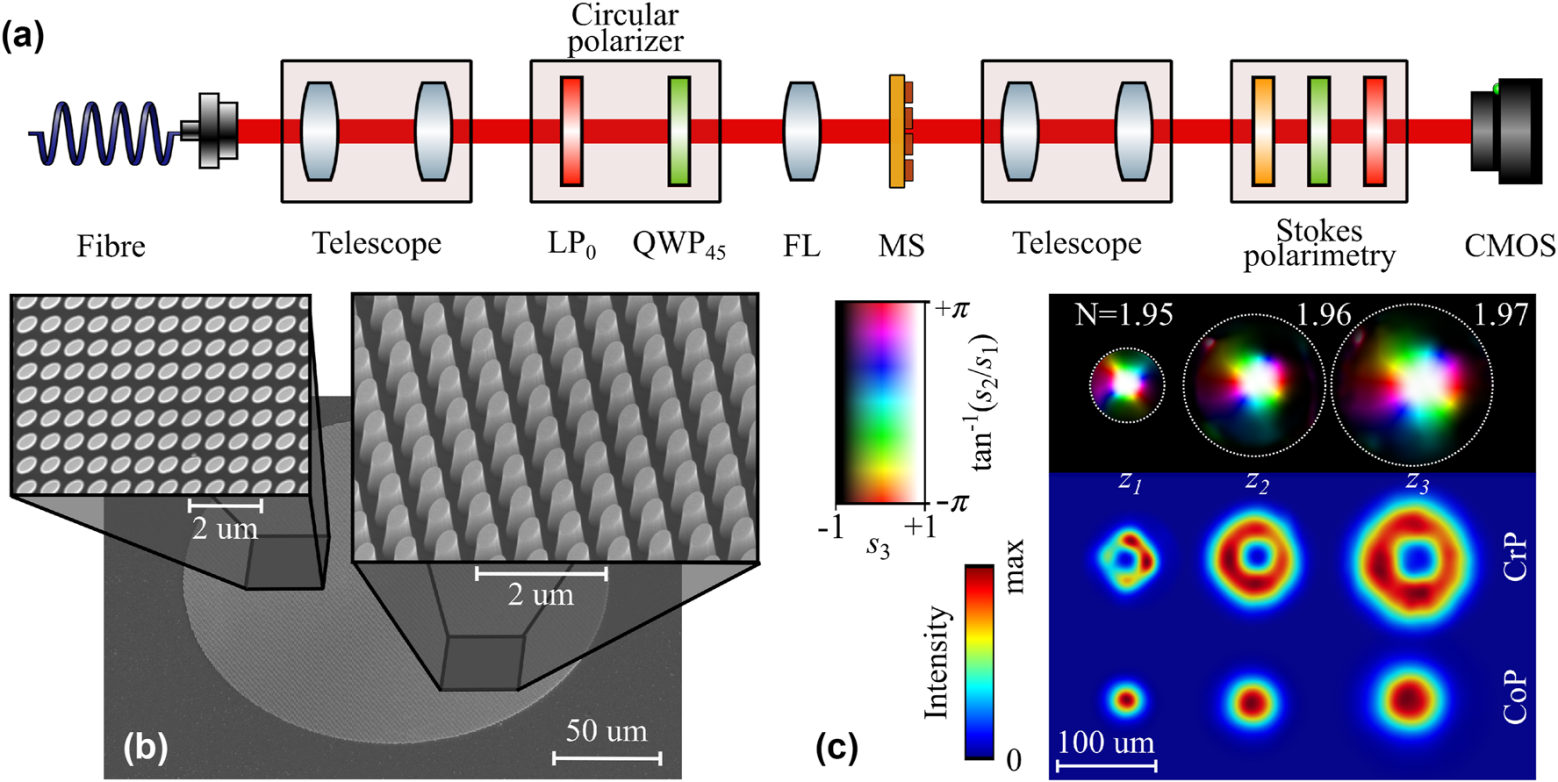
Experimental results. (a) Experimental setup for polarization characterization of Stokes skyrmions. LP: linear polarizer, QWP: quarter-wave plate, FL: focusing lens, MS: metasurface. (b) Scanning electron microscope (SEM) images of the fabricated MS for top and tilted views. (c) Experimentally measured Stokes texture (top row) for three different diffraction distances measured from the image plane of the telescope (*z*_3_ = 0 mm, *z*_2_ = 0.12 mm, *z*_1_ = 0.72 mm) with the calculated skyrmion number. The lower rows show the corresponding intensity profile of the CrP and CoP waves.

We present a first preliminiary proof-of-concept experimental realisation of our approach. First, a layer of 800-nm aSi was deposited on a quartz substrate via plasma-enhanced chemical vapor deposition (Oxford PlasmaPro100). PMMA A4 resist was then spin-coated at 3,000 rpm for 1 min, followed by soft baking at 180 °C for 3 min on a hotplate. The designed MS pattern was defined using a 100 kV EBL system (Raith EBPG). After development, a 30-nm Chromium mask layer was deposited onto the sample in an E-beam evaporation chamber (Intlvac Nanochrome II) under 5 · 10^−6^ Torr. Excess PMMA and Cr were removed by immersing the sample in acetone and rinsing it in IPA. The mask pattern was then transferred to the a-Si layer by ICP-RIE (Oxford PlasmaLab) dry etching, using a mixture of SF_6_ and C_4_F_8_ gases for the reactive and physical etch processes, respectively. The SEM images of the fabricated MSs for different magnifications and view angles are shown in Figure 5(b). Once the samples are fabricated, we prepare an imaging setup for polarization characterization as shown in 5(a). A fibre-coupled 1,550 nm Gaussian beam is expanded and collimated with a telescope. We prepare the input circular polarization impinging on the MS by a linear polarizer at 0° and quarter-wave plate at 45°. Then, the beam is focused onto a spot of FWHM ∼60 μm at the MS plane using a lens. The output beam is imaged using a telescope and captured by a NIR CMOS detector (NINOX 640 II). We perform standard Stokes polarimetry, in which the polarization features are retrieved from six intensity measurements in different polarization basis (H,V,D,A,L,R)

(7) $$\begin{array}{cc} s_{1} & =\left(H-V\right)/\left(H+V\right),\\ s_{2} & =\left(D-A\right)/\left(D+A\right),\\ s_{3} & =\left(L-R\right)/\left(L+R\right). \end{array}$$

Finally, in 5(c) we depict the reconstructed skyrmion textures (top row) at three different propagation distances with their corresponding skyrmion numbers. The distances (*z*_3_ = 0 mm, *z*_2_ = 0.12 mm, *z*_1_ = 0.72 mm) are measured respect to the image plane of the telescope. The CrP and CoP intensity profiles are also shown in the lower rows, whose diffraction evolution mainly features a spatial stretching without significant shape changes, up to experimental and fabrication errors. As a corollary, the skyrmion topology remains preserved upon propagation in free-space, maintaining a stable skyrmion number across the measured diffraction distances. We remark that this is a consequence of achieving a constant dynamic phase along the MS plane, which is confirmed by interferometric measurements shown in Figure S1 of SI. Due to the small size of the MS (*D* ∼200 μm) preparing a collimated illumination at the MS plane constitutes experimental challenges, which complicates the characterization of the output fields within their Rayleigh range as was theoretically done in 4(d). In our experiments due to non-ideal collimation the input beam included a focusing wavefront causing the generated skyrmions to converge. This will be improved by increasing the MS size, and a more precise characterization of the topological features will be carried out in future works.

## 4 Conclusions

In summary, we have designed a dielectric MS based on structural birefringence and geometric phase which achieves arbitrary CoP to CrP conversion without carrying any additional dynamic phase. This design empowered by the modulation of light at dynamic phase iso-curves facilitates the generation of scalar modes that align closely with free-space eigenmodes, avoiding additional wavefronts typically associated with the dynamic phase. We have numerically and experimentally verified that the resulting light fields feature a nearly invariant evolution during propagation, undergoing only a spatial stretching in their diffraction profiles and guaranteeing the stability of the skyrmion topology across propagation distances. Our findings show significant advancements in the field, presenting a compact platform for generating topological structured light with stable topological features, which may open new pathways for applications in optical information transfer and other technologies reliant on topological structured light.

## Supplementary Material

Supplementary Material Details
