# Supplementary material for: Tailoring propagation-invariant topology of optical skyrmions with dielectric metasurfaces
Source: Nanophotonics. 2025 Mar 13;14(23):4069–77. doi: 10.1515/nanoph-2024-0736 (PMC12617712; doi:10.1515/nanoph-2024-0736)
Supplement: Supplementary file 1 — Supplementary Material Details [file j_nanoph-2024-0736_suppl_001.pdf]

Supplementary Material for Tailoring Propagation-Invariant Topology of Optical Skyrmions with Dielectric Metasurfaces

S1. JONES MATRIX RESPONSE OF THE METASURFACE

The response of the MS can be expressed by the product of its Jones matrix for an arbitrary in-plane rotation angle θ with the input polarization state, which for LCP illumination reads

$$\boldsymbol{E}_{\text{out}} = \mathcal{T}(\theta)\cdot\boldsymbol{E}_{\text{in}} = \begin{bmatrix}\cos\theta & -\sin\theta\\ \sin\theta & \cos\theta\end{bmatrix}\cdot\begin{bmatrix}t_{xx} & 0\\ 0 & t_{yy}\end{bmatrix}\cdot\begin{bmatrix}\cos\theta & \sin\theta\\ -\sin\theta & \cos\theta\end{bmatrix}\cdot\frac{1}{\sqrt{2}}\begin{bmatrix}1\\ i\end{bmatrix}.$$

The output state is decomposed in terms of the CoP (LCP) and CrP (RCP) components, which after some elementary algebra it simplifies to

$$\boldsymbol{E}_{\text{out}} = \frac{1}{2}(t_{xx}+t_{yy})\boldsymbol{u}_L + \frac{1}{2}(t_{xx}-t_{yy})e^{i2\theta}\boldsymbol{u}_R.$$

We can further simplify the description of the transmitted polarization state by considering that the Jones matrix is unitary, i.e. $t_{xx} = e^{i\phi_x}$, $t_{yy} = e^{i\phi_y}$. We define the degree of birefringence (DOB) of the MS through the phase difference between the principal axes transmittances:

$$\text{DOB}\,(\Delta\phi) = \sin\frac{\Delta\phi}{2} = \sin\frac{\phi_y-\phi_x}{2},$$

which ranges from 0, isotropic plate, to 1, half-wave plate (HWP), for increasing phase difference between 0 and π. The transmission through the MS in this simplified unitary approach reads

$$\boldsymbol{E}_{\text{out}}(\boldsymbol{r}) = e^{i\xi(\boldsymbol{r})}\left(\cos\left(\frac{\Delta\phi(\boldsymbol{r})}{2}\right)\boldsymbol{u}_L + i\sin\left(\frac{\Delta\phi(\boldsymbol{r})}{2}\right)e^{i2\theta(\boldsymbol{r})}\boldsymbol{u}_R\right), \tag{S1}$$

where we have made explicit that all the magnitudes (ξ, $\Delta\phi$, θ) are position-dependent. A global dynamic phase factor $\xi = (\phi_y+\phi_x)/2$ appears in both polarization components due to the propagation through the MS. By tuning the DOB of the meta-atoms, one can modulate the amplitude of the CrP light independently from its phase, which is controlled by the in-plane rotation angle θ through the geometric phase. This kind of MSs allows us to have full control of the Stokes parameters (S_1, S_2, S_3)

$$S_1 = 2\mathcal{R}e\{\psi_l^*\psi_r\},\quad S_2 = -2\mathcal{I}m\{\psi_l^*\psi_r\},\quad S_3 = |\psi_l|^2 - |\psi_r|^2,$$

where $\mathcal{R}e\{\cdot\}$ and $\mathcal{I}m\{\cdot\}$ denote real and imaginary parts respectively. From the complex field (S1) we can derive the Stokes parameters as

$$S_1 = \sin(\Delta\phi)\cos\left(2\theta+\frac{\pi}{2}\right),\qquad S_2 = -\sin(\Delta\phi)\sin\left(2\theta+\frac{\pi}{2}\right),\qquad S_3 = \cos(\Delta\phi).$$

Note that the resulting vector is normalized,

$$S_1^2+S_2^2+S_3^2 = 1,$$

courtesy of the unitarity of the Jones matrix. It is useful to define the Stokes vector in a polar basis, where the new coordinates are the latitude $\mathcal{L} = S_3$ and the azimuth $\mathcal{Z} = \arctan(S_2/S_1)$ in the PS, being reduced to the simple expression

$$\mathcal{L} = \cos(\Delta\phi),\qquad \mathcal{Z} = \frac{\pi}{2} - 2\theta. \tag{S2}$$

S2. WAVEFRONT COMPENSATION

To give a simple physical perspective, we can describe the phase accumulation through the principal axes of the MS using the plane-wave expressions:

$$\phi_x = k_0 \cdot n_x^{eff} \cdot \Delta z, \qquad \phi_y = k_0 \cdot n_y^{eff} \cdot \Delta z,$$

with k_0 the vacuum wavenumber, Δz the height of the meta-atoms and n_x^{eff}, n_y^{eff} their effective refractive indices along the x and y axes respectively. For a given wavelength and height, the polarization response (S1) of the MS is determined by the effective indices

$$\Delta\phi = k_0 \cdot \Delta z \left(n_y^{eff} - n_x^{eff}\right), \qquad \xi = \frac{1}{2} k_0 \cdot \Delta z \left(n_y^{eff} + n_x^{eff}\right).$$

Without rotation θ, the difference between the two refractive indices determines the amplitude of the two waves, while the sum of them determines the shape of the wavefront ξ. To explain the amplitude modulation we start from the case where the meta-atoms are symmetric ($n_y^{eff} = n_x^{eff} = n_0$), and the LCP incident light simply yields LCP transmitted light with a phase factor $\xi_0 = k_0 \cdot \Delta z \cdot n_0$. If we now increase slightly the value of n_y^{eff} to n_{y1}^{eff}, we will convert part of the incident LCP light into RCP in transmission, but the phase acquired will be different with respect to the isotropic phase $\xi_1 - \xi_0 = \frac{1}{2} k_0 \cdot \Delta z \left(n_{y1}^{eff} - n_0\right)$. However, we can now decrease the value of the refractive indices of both axes in the same amount $\delta n/2$

$$n_{y2}^{eff} = n_{y1}^{eff} - \delta n/2, \qquad n_{x2}^{eff} = n_0 - \delta n/2$$

keeping their difference intact ($n_{y2}^{eff} - n_{x2}^{eff} = n_{y1}^{eff} - n_0$), and so the polarization conversion efficiency. Choosing $\delta n/2$ with the constraint $\delta n = n_{y1}^{eff} - n_0$ the phase accumulation will be exactly the same as that of the isotropic meta-atoms, thus the polarization conversion carries no additional phase change. Repeating the procedure again until we reach $\Delta\phi = \pi$ will allow full control of the CrP amplitude. In practice, the modulation of the effective refractive indices is carried out by changing the polarizability of the meta-atoms in two orthogonal axes, for example using an elliptical cross section and tuning the length of the two diameters. Simultaneous reduction of the effective refractive indices of the meta-atoms along the two principal axes is necessary to compensate the dynamic phase and thus achieving a flat CoP wavefront. A preliminary simulation is carried out using gRCWA [1] at the telecommunications wavelength $\lambda = 1.55\,\mu m$. We have chosen a-Si meta-atoms with elliptical cross-section and constant height $h = 800\,nm$ laying on top of a quartz (SiO2) substrate. The unit cell is squared with array pitch $P = 650\,nm$. At first, we simply sweep the two principal diameters of the ellipses (D_1, D_2) in a range where we can achieve maximal polarization conversion efficiency, from 210 nm to 535 nm. The input light is circularly polarized (LCP) and the output light

$$\boldsymbol{E}_{\text{out}} = E_x \boldsymbol{u}_x + E_y \boldsymbol{u}_y = \frac{1}{\sqrt{2}}(E_x - iE_y)\boldsymbol{u}_L + \frac{1}{\sqrt{2}}(E_x + iE_y)\boldsymbol{u}_R$$

is decomposed into the CoP (LCP) and CrP (RCP) components, which are two different complex numbers (amplitude and phase).

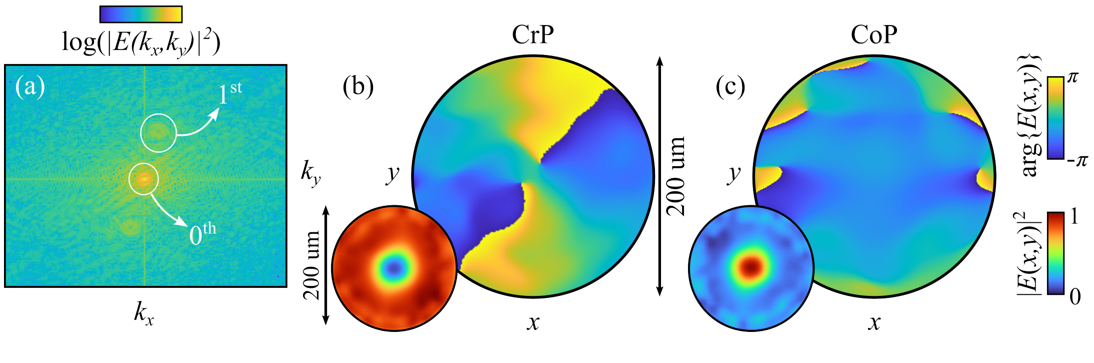

FIG. S1: Experimental measurements of the CoP and CrP phase profiles. (a) Logaritmic plot of the Fourier-transformed interference patterns between reference and output beams showing first and zeroth interference orders. Phase and intensity (inset) profiles at the MS plane of CrP and CoP waves are shown in (b) and (c) respectively.

The experimental demonstration of polarization manipulation at dynamic phase iso-lines is confirmed by measuring the wavefront of CoP and CrP waves. We carry out standard interferometric measurements with fringe analysis using the setup shown in Fig. 5 of main text introducing a tilted reference beam after the Stokes polarimetry. The interference fringes are then Fourier transformed and the phase is retrieved by cropping the first interference order [2]. The results are shown in Fig. S1: (a) shows the Fourier transform of the interference fringes, where the first order carries the information of the phase. By selecting RCP/LCP with the Stokes polarimeter and its corresponding first order in (a) we can extract the phase of CrP and CoP waves, which is shown in (b) and (c) respectively along with their intensity profiles. The experimental results show remarkable flat wavefronts (except for the vortex phase in (b)), which confirms the expected theoretical results.

[1] W. Jin, W. Li, M. Orenstein, and S. Fan, ACS Photonics **7**, 2350 (2020).
[2] Z. Malacara and M. Servin, Interferogram analysis for optical testing (CRC press, 2018).
